# Editorial: APPNING: Animal Population Imaging

**DOI:** 10.3389/fninf.2021.676603

**Published:** 2021-05-13

**Authors:** Michel Dojat, Jan G. Bjaalie, Emmanuel L. Barbier

**Affiliations:** ^1^University Grenoble Alpes, Inserm U1216, Grenoble Institut Neurosciences, Grenoble, France; ^2^Department of Molecular Medicine, Institute of Basic Medical Sciences, University of Oslo, Oslo, Norway

**Keywords:** data sharing, open science, neuroimaging, brain, neuroscience

This editorial review of the Research Topic Appning describes several solutions to support the sharing of animal imaging data and processing tools. Appning promotes the federation of multiple sources of information, processing tools and shows how this contributes to the diffusion of knowledge distributed in various preclinical imaging centers.

## Animal Data Sharing

Some large data repositories (e.g., for brain studies, The MJ Fox Parkinson's database or the Human Connectome project) and specific architectures (e.g., COINS, LONI) are now available for human population imaging. The animal imaging community has also growing requirements for multicenter studies, for example to allow the comparison of academic results as in brain connectivity studies (Grandjean et al., 2020) or to characterize the effects of drugs (Bruns et al., 2015). To share preclinical imaging data and data analysis pipelines, only few tools are available that take into account the specificities of animal studies (Liu et al., 2020; Messinger et al., 2020), and few studies aim at standardization of acquisition and post-processing techniques.

Kain et al. describe a solution, *Small Animal Shanoir* (*SAS*), for the management of imaging data and metadata. *SAS* is a preclinical extension of a cloud-based solution dedicated to the management of human brain imaging repositories, Shanoir (Barillot et al., 2016). The main feature of this working solution is to rely on a core ontology, OntoNeurolog, which allows for the federation of different local databases via the mapping of their corresponding data models to the ontology, and facilitates its extension, for instance for managing preclinical studies. Additionally, to reinforce its extensibility capacity, *SAS* is designed as a set of independent micro-services. Then, a specific micro-service, *Dicomifier*, is dedicated to the transformation to Nifti format, widely used by several neuroimaging pipelines, of raw files in Bruker format, Bruker being a manufacturer of preclinical MR scanners, or of Dicom files. Associated data acquisition parameters are kept under a json file associated to each Nifti file stored in SAS. The web-oriented architecture allows for querying and retrieving stored images and processing pipelines. A data transfer module can interface the data management system to computing platforms for pipelines execution and the storage of image processing results. Specific authentication mechanisms allow for the fine control of data access, from an access restricted to a specific user's community to a publicly access for promoting open science. *SAS* was used for the project described by Deruelle et al. in this Research Topic.

Mandino et al. review the efforts done by the animal MRI community toward the standardization of data acquisition and analysis procedures in the context of whole brain functional MRI; a key aspect for animal population imaging via multi-center studies. Based on their 868 research papers analysis, they showed that animal studies (mainly on rats and Sprague Dawley strain, in general carried out on 10 subjects at 7T and 9.4T) were underpowered and the false-positive rate incorrectly controlled, similarly to human studies (Button et al., 2013; Eklund et al., 2016). Several sources of variations among studies, from animal preparation and anesthesia to the use of *ad-hoc* pipelines or/and *ad-hoc* templates, hamper the comparison of published results. The authors propose guidelines to improve data sharing and reproducibility. They emphasize the importance of raw datasets sharing for data re-analysis with other processing pipelines allowing results comparison between studies, the adoption of standard templates for the reporting of results (e.g., coordinates of activation clusters) similarly to human neuroimaging studies (Fox et al., 2014), and the availability of open-source validated pipelines to unify data processing.

## Pipelines Composition and Pipeline Sharing

As shown by Mandino et al. in their review of the small animal literature, a source of difficulty for comparing results in animal studies is the absence of a core of validated solutions for data processing and analysis, similarly to what is available for human neuroimaging. Four papers address this point. To facilitate the sharing of raw MR brain imaging data, Ioanas et al. present a tool to transform files in a proprietary format (Bruker files) to Bids format that has been proposed for human neuroimaging studies (Gorgolewski et al., 2016). The workflow is implemented as a function, *bru2bids*, written in Python. In the same vein, Celestine et al. propose the Python package *Samba-MRI* to preprocess, register to templates, perform functional analysis, and perfusion measures from raw MR brain imaging datasets. It reuses several neuroimaging python libraries (e.g., Nipype, Nibabel or Nilearn) and incorporates additional features for group-wise registration or inter-modality registration. The code is available via the open GitHub platform. One can mention here the recent work of Brossard et al. (2020) who introduce a package to design pipelines and obtain multiparametric MRI maps that was extensively evaluated at the preclinical level. The papers from Groeneboom et al. and Yates et al. propose software for analyzing histological rodent brain images. Indeed, the recent *in vitro* imaging systems provide large collections of high-resolution images that raise specific computational problems for memory management and time execution. The former, *Nutil*, allows to automatize image processing and analysis of 2D brain histological sections. Standard image transformations are proposed to the user and their execution has been optimized to deal with large datasets. *Nutil* can be used independently or conjointly with the *Quint* workflow. *Quint* is a suite of tools that allows for the quantification and the spatial analysis of selected features in series of histological section images of rodent brain within a known atlas space. It combines several pre-existing tools for pre-processing, registration to 3D reference atlas (mouse and rat) and object segmentation, for the quantification of specific parameters in regions defined by the atlas. The *Quint* suite allows the user to perform in a convenient way, a quantitative analysis at different levels of granularity on large imaging datasets.

## Applications of Existing Data Sharing and Data Analysis Solutions

MR imaging is a non-invasive versatile technique that allows to assess to various anatomical, functional or physiological parameters. Then, the T1 and T2 relaxation times are tissue and region-dependent parameters that may reflect structural alterations and may be used as biomarkers for various pathologies. The goal of Deruelle et al. was to define maps of T1 and T2 values for specific rat brain regions. To serve as reference, such maps should be defined based on a sufficient number of healthy animals reflecting inter-individual variability. They designed a multi-center study which included two data provider centers and three image processing pipeline provider centers. They used the SAS architecture (Kain et al.) and showed first, that MR data from 40 rats acquired in two centers could be successfully combined; second, that a good reproducibility could be obtained when using different processing solutions. Their study demonstrates the feasibility of a multi-center animal study if an appropriate architecture for data management and pipelines composition and execution is available. Raw data and reference T1 and T2 relaxometry maps, as well as processing pipelines are freely available via SAS.

Badea et al. use genetically modified mouse models to relate genotype with brain aging trajectory for Alzheimer's disease. They use diffusion tensor imaging to compare structural connectomes, and region volumes for two gene modified mouse groups associated with genetic risk for Alzheimer disease. Additionally, behavioral tests provide information about learning and memory function deficits. They show that behavioral and imaging markers allow to identify vulnerable brain networks induced by the genetic risk factor. Their findings contribute to a better understanding of the physio-pathological mechanisms triggering the onset of the Alzheimer's disease. All generated datasets generated are available to the scientific community and may be pooled with new data to reinforce the robustness of the findings.

Interestingly, all the papers of the RT Appning concern the investigation of the rodent brain with both *in vivo*, MR imaging, and *in vitro*, histological imaging. Indeed, neuroimaging is the domain for which scientists have developed to date several solutions for sharing data and process large data repositories. This is certainly due to the impressive quantity of neuroimaging studies performed in the recent years, generating large-scale databases. This is also stimulated by publications showing the poor replicability and reproducibility of the results obtained (Carp, 2012; Button et al., 2013; Nichols et al., 2017; Poldrack et al., 2017). Because these caveats are not restricted to brain studies, but concern many areas of life science (Ioannidis, 2005), new solutions for dealing with other organs and species will for sure emerge.

## Author Contributions

MD wrote the first draft of the manuscript. All authors contributed to manuscript revision, read, and approved the submitted version.

## Conflict of Interest

The authors declare that the research was conducted in the absence of any commercial or financial relationships that could be construed as a potential conflict of interest.
